# Discrimination of Temperature and Strain in Brillouin Optical Time Domain Analysis Using a Multicore Optical Fiber

**DOI:** 10.3390/s18041176

**Published:** 2018-04-12

**Authors:** Mohamed A. S. Zaghloul, Mohan Wang, Giovanni Milione, Ming-Jun Li, Shenping Li, Yue-Kai Huang, Ting Wang, Kevin P. Chen

**Affiliations:** 1 Department of Electrical and Computer Engineering, University of Pittsburgh, Pittsburgh, PA 15261, USA; mow10@pitt.edu; 2 Optical Networking and Sensing Department, NEC Laboratories America, Inc., Princeton, NJ 08540, USA; gmilione@nec-labs.com (G.M.); kai@nec-labs.com (Y.-K.H.); ting@nec-labs.com (T.W.); 3 Corning Research and Development Corporation, Corning, NY 14831, USA; lim@corning.com (M.-J.L.); lis2@corning.com (S.L.)

**Keywords:** fiber optics sensors, Brillouin Scattering, optical time domain reflectometry, fiber characterization

## Abstract

Brillouin optical time domain analysis is the sensing of temperature and strain changes along an optical fiber by measuring the frequency shift changes of Brillouin backscattering. Because frequency shift changes are a linear combination of temperature and strain changes, their discrimination is a challenge. Here, a multicore optical fiber that has two cores is fabricated. The differences between the cores’ temperature and strain coefficients are such that temperature (strain) changes can be discriminated with error amplification factors of 4.57 °C/MHz (69.11 μϵ/MHz), which is 2.63 (3.67) times lower than previously demonstrated. As proof of principle, using the multicore optical fiber and a commercial Brillouin optical time domain analyzer, the temperature (strain) changes of a thermally expanding metal cylinder are discriminated with an error of 0.24% (3.7%).

## 1. Introduction

Brillouin optical time domain analysis (BOTDA) is the sensing of temperature and strain changes along an optical fiber by measuring the frequency shift changes of Brillouin backscattering. As compared to other sensing modalities (e.g., Rayleigh- and Raman-based), BOTDA is not strictly power dependent and can be used over distances as long as 100 km. BOTDA may be used to monitor the operation and structural integrity of oil and gas pipelines and wells, electrical power lines, and transportation infrastructure (e.g., bridges, railroads, highways) [1,2].

Because frequency shift changes are a linear combination of temperature and strain changes, their discrimination is a challenge [3,4,5]. Temperature and strain can be discriminated by solving a system of linear equations that comprise measurements of frequency shift changes from two spatial channels (spatial channels 1 and 2). The spatial channels are subjected to the same temperature and strain changes, yet they have different temperature and strain coefficients. For example, two single-mode optical fibers can be used [6]. However, because they occupy different claddings, they may not experience the same temperature and strain changes. Additionally, two spatial modes of a multimode optical fiber can be used [7,8,9]. However, because they occupy the same core, the multimode optical fiber may not be fabricated such that the coefficients are sufficiently different. Another solution using two fibers in a cable to discriminate temperature and strain has been proposed [10], in which one fiber is deployed in a loose tube to measure the temperature only, and the other is deployed in a tight buffer to sense both the temperature and strain. However, this approach has two issues that cause measurement errors. Firstly, the loose-tubed fiber is not completely strain-free, which results in measurement errors on temperature and strain. Secondly, the difference in fiber length between the two fibers also causes measurement errors at sensing locations. 

Recently, it was demonstrated that two cores of a multicore optical fiber can be used [11,12]. This contrasts the use of two single-mode optical fibers and two spatial modes of a multimode optical fiber. Because the cores occupy the same cladding, they can experience the same temperature and strain changes. Additionally, because the cores are separate, the multicore optical fiber can be fabricated such that the coefficients are sufficiently different. In general, the ability of spatial channels to discriminate temperature from strain is quantified by error amplification factors, which are a measure of the coefficients’ differences [13]. For multicore optical fibers, temperature (strain) error amplification factors greater than 12.05 °C/MHz (253.77 μϵ/MHz) were demonstrated. Therefore, it may be of interest to fabricate a multicore optical fiber such that the error amplification factors are lower.

Here, a multicore optical fiber that has two cores is fabricated. The differences between the cores’ temperature and strain coefficients are such that temperature(strain) changes can be discriminated with error amplification factors of 4.57 °C/MHz (69.11 μϵ/MHz), which is 2.63 (3.67) times lower than previously demonstrated. As proof of principle, using the multicore optical fiber and a commercial BOTDA, the temperature (strain) changes of a thermally expanding metal cylinder are discriminated with an error of 0.24% (3.7%).

## 2. Theory 

In BOTDA, frequency shift changes are a linear combination of temperature and strain changes. Temperature and strain changes can be discriminated by solving a system of linear equations that comprise measurements of frequency shift changes from two spatial channels (spatial channels 1 and 2). The spatial channels are subjected to the same temperature and strain changes, yet they have different temperature and strain coefficients. In matrix form, where the coefficients comprise a matrix, the system of linear equations is given by [13]:

(1) $$\left[\begin{array}{c} \Delta \nu_{1}+\delta \nu \\ \Delta \nu_{2}+\delta \nu \end{array}\right]=\left[\begin{array}{cc} c_{T,1} & c_{\epsilon ,1}\\ c_{T,2} & c_{\epsilon ,2} \end{array}\right]\;\left[\begin{array}{c} \Delta T\\ \Delta \epsilon \end{array}\right]$$

$\Delta \nu_{1}$ and $\Delta \nu_{2}$ are the measured frequency shifts changes of spatial channels 1 and 2, respectively. δν is the measurement error of $\Delta \nu_{1}$ and $\Delta \nu_{2}$. It is assumed that δν is the same for $\Delta \nu_{1}$ and $\Delta \nu_{2}$, because it is assumed that $\Delta \nu_{1}$ and $\Delta \nu_{2}$ are measured in the same manner. $c_{T,1}$ and $c_{\epsilon ,1}$ ($c_{T,2}$ and $c_{\epsilon ,2}$) are the temperature and strain coefficients, respectively, of spatial channel 1 (spatial channel 2). ΔT and Δϵ are the temperature and strain changes, respectively, experienced by spatial channels 1 and 2. $c_{T,1}$ and $c_{T,2}$ ($c_{\epsilon ,1}$ and $c_{\epsilon ,2}$) can be experimentally determined by measuring $\Delta \nu_{1}$ and $\Delta \nu_{2}$ while varying ΔT (Δϵ) and keeping Δϵ (ΔT) constant. Effectively, $c_{T,1}$, $c_{T,2}$, $c_{\epsilon ,1}$ and, $c_{\epsilon ,2}$ are the slopes of best fit lines of the measurements. It is assumed that the measurement errors of $c_{T,1}$, $c_{T,2}$, $c_{\epsilon ,1}$ and, $c_{\epsilon ,2}$ are negligible. ΔT and Δϵ can be solved for by multiplying both sides of Equation (1) by the inverse of the matrix. It can be shown that the resulting errors of ΔT and Δϵ are given, respectively, by the equations [13]:

(2) $$\delta T=\delta \nu \frac{\sqrt{{\left(c_{\epsilon ,2}\right)}^{2}+{\left(c_{\epsilon ,1}\right)}^{2}}}{c_{T,1}c_{\epsilon ,2}-c_{\epsilon ,1}c_{T,2}},$$

and,

(3) $$\delta \epsilon =\delta \nu \frac{\sqrt{{\left(c_{T,2}\right)}^{2}+{\left(c_{T,1}\right)}^{2}}}{c_{T,1}c_{\epsilon ,2}-c_{\epsilon ,1}c_{T,2}}.$$

Note that Equations (2) and (3) were calculated in accordance with error propagation, i.e., all terms that comprise δν were added in quadrature. As can be seen, $c_{T,1}$, $c_{T,2}$, $c_{\epsilon ,1}$ and, $c_{\epsilon ,2}$ make up factors that multiplicatively increase (amplify) δν. These factors are referred to here as temperature and strain error amplification factors, respectively, i.e., δT/δv and δϵ/δv, respectively. Effectively, δT/δv and δϵ/δv are a measure of the difference between $c_{T,1}$, $c_{T,2}$, $c_{\epsilon ,1}$ and, $c_{\epsilon ,2}$. The ability of the spatial channels to discriminate ΔT and Δϵ is quantified by δT/δv and δϵ/δv, respectively. 

## 3. Experiment

### 3.1. Multicore Optical Fiber

**Figure 1:**
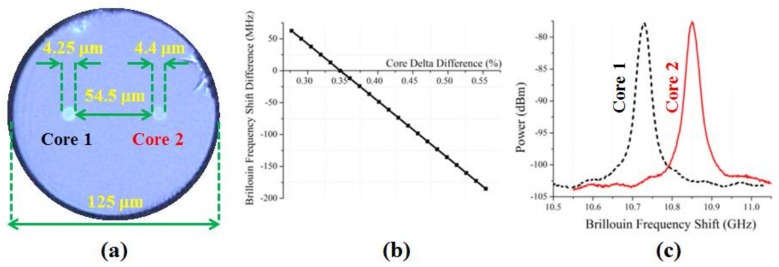
(**a**) Cross-section of multicore optical fiber that has two cores (cores 1 and 2) as described in the text; (**b**) theoretical calculation of the difference between the frequency shifts of cores 1 and 2 as a function of the difference between their deltas as described in the text; (**c**) measured frequency shifts of cores 1 and 2 as described in the text.

A multicore optical fiber with two cores (core 1 and 2) was fabricated. Details regarding the transmission characteristics of cores 1 and 2 can be found in references [14,15]. The transmission characteristics of cores 1 and 2 were comparable to those of a standard single-mode optical fiber; however, because the fiber was made in a research lab, the attenuation (0.23 dB/km for Core 1, and 0.29 dB/km for Core 2) was slightly higher than that of a standard single-mode fiber. It is expected that the attenuation will decrease when the fiber is made in the production process. The fiber diameter (9.3 μm for Core 1, and 10.4 μm for Core 2) is similar to the standard single-mode fiber diameter, and therefore compatible for typical Brillouin sensing applications. Figure 1a shows the multicore optical fiber’s cross-section. It had a 125 μm diameter cladding. Cores 1 and 2 served as spatial channels 1 and 2, respectively. Cores 1 and 2 had radii of 4.25 μm and 4.4 μm, respectively, and were separated by 54.5 μm. Cores 1 and 2 were Ge doped, had step index profiles, and had deltas of 0.46% and 0.34%, respectively. Figure 1b shows a theoretical calculation of the difference between the frequency shifts of cores 1 and 2 as a function of the difference between their deltas when one of the cores had a delta of 0.34% [14]. The frequency shifts from cores 1 and 2 were measured using a commercial BOTDA. The commercial BOTDA is described below. Figure 1c shows the measured frequency shifts, which were 10.73 GHz and 10.85 GHz, respectively. Using different core compositions, the fiber cores’ responses to temperature and strain (i.e., $c_{T,1}$, $c_{T,2}$, $c_{\epsilon ,1}$ and, $c_{\epsilon ,2}$) were also sufficiently different.

### 3.2. Commercial Brillouin Optical Time Domain Analyzer

In the experiments, $\Delta \nu_{1}$ and $\Delta \nu_{2}$ were measured using a commercial BOTDA (OZ Optics). A light pulse (1550 nm) was launched into cores 1 or 2 at one end of the multicore optical fiber, and continuous-wave light was launched into cores 1 or 2, respectively, at the other end (i.e., counter propagating pulse and continuous-wave). The light pulse and continuous-wave light interact via an electrostriction-induced acoustic wave, i.e., stimulated Brillouin back-scattering. The frequency difference between the light pulse and the continuous wave light could be varied from 10.5 GHz to 11.0 GHz. Due to stimulated Brillouin scattering, there was power amplification (gain) when the frequency shift of the spontaneous Brillouin back-scattering was equal to the frequency difference between the light pulse and the continuous wave light. $\Delta \nu_{1}$ and $\Delta \nu_{2}$ were measured by measuring that power amplification (i.e., the Brillouin gain spectrum). The spatial resolution of the commercial BOTDA was 1 m. The lengths of the multicore optical fibers used in the experiments were 20 m. $\Delta \nu_{1}$ and $\Delta \nu_{2}$ were measured by measuring a frequency shift every 1 m over 20 m and then averaging. δν of the commercial BOTDA depended on many factors, including launched power, optical fiber length, and measurement time. In the experiments, δν was ~0.64 MHz. 

The commercial BOTDA had two channels (channels 1 and 2). $\Delta \nu_{1}$ and $\Delta \nu_{2}$ were measured simultaneously by connecting cores 1 and 2 to channels 1 and 2. Cores 1 and 2 were connected to channels 1 and 2, respectively, via fusion splicing of a single-mode optical fiber. However, due to size, only one single-mode optical fiber could be spliced to a core at a time. Therefore, two multicore optical fibers were used: One single-mode optical fiber was spliced to core 1 of one multicore optical fiber, and another single-mode optical fiber was spliced to core 2 of another multicore optical fiber.

### 3.3. Experimental Determination of $c_{T,1}$ and $c_{T,2}$

**Figure 2:**
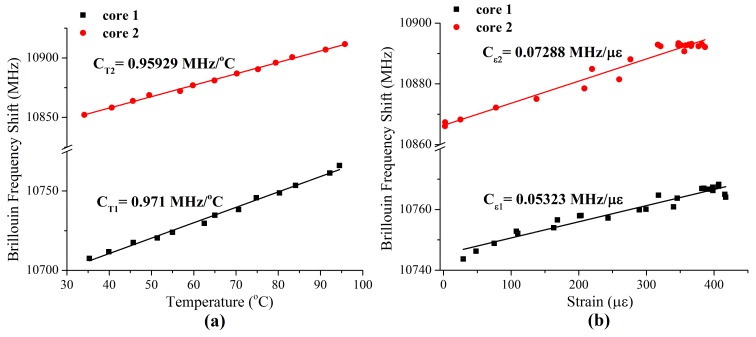
(**a**) Experimental determination of temperature coefficients ($c_{T,1}$ and $c_{T,2}$)—plots and best fit lines of $\Delta \nu_{1}$ and $\Delta \nu_{2}$ as a function of ΔT as described in the text; (**b**) experimental determination of strain coefficients ($c_{\epsilon ,1}$ and $c_{\epsilon ,2}$)—plots and best fit lines of $\Delta \nu_{1}$ and $\Delta \nu_{2}$ as a function of Δϵ as described in the text.

$c_{T,1}$ and $c_{T,2}$ were experimentally determined by measuring $\Delta \nu_{1}$ and $\Delta \nu_{2}$ while varying ΔT and keeping Δϵ constant. Effectively, $c_{T,1}$ and $c_{T,2}$ are the slopes of best fit lines of the measurements. The 20 m lengths of the two multicore optical fibers were loosely coiled and placed in an oven. Note that loose coiling ensured that Δϵ was constant. ΔT was varied by varying the temperature of the oven. The temperature of the oven was varied from 35 °C to 95 °C in increments of 5 °C. A thermocouple was also placed in the oven. The thermocouple was used to measure the temperature of the oven. For each increment, $\Delta \nu_{1}$ and $\Delta \nu_{2}$ were measured using the commercial BOTDA. $\Delta \nu_{1}$ and $\Delta \nu_{2}$ were plotted as a function of ΔT. Figure 2a shows the plots. $c_{T,1}$ and $c_{T,2}$ were determined by calculating the best fit lines of the plots. Figure 2a also shows the plots of the best fit lines. The determined values of $c_{T,1}$ and $c_{T,2}$ are shown in the first row of Table 1. The measurement errors of $c_{T,1}$ and $c_{T,2}$ were 0.02456 MHz/°C and 0.01456 MHz/°C, respectively.

### 3.4. Experimental Determination of $c_{\epsilon ,1}$ and $c_{\epsilon ,2}$

$c_{\epsilon ,1}$ and $c_{\epsilon ,2}$ were experimentally determined by measuring $\Delta \nu_{1}$ and $\Delta \nu_{2}$ while varying Δϵ and keeping ΔT constant. Effectively, $c_{\epsilon ,1}$ and $c_{\epsilon ,2}$ are the slopes of best fit lines of the measurements. The two 20 m lengths of the multicore optical fiber were wound between two posts that were separated by 10 m. The position of one post was fixed and the position of the other post was varied by a motor controlled stage (Thorlabs^®^). The motor-controlled stage had 0.1 μm and 8 μm position resolution and repeatability, respectively. Δϵ was varied by varying the position of the motor-controlled stage that stretched the two 20 m lengths of the multicore optical fiber. Δϵ was varied from 0 μϵ to 400 μϵ in various increments. The determination of the increments is described below. For each increment, $\Delta \nu_{1}$ and $\Delta \nu_{2}$ were measured using the commercial BOTDA. $\Delta \nu_{1}$ and $\Delta \nu_{2}$ were plotted as a function of Δϵ. Figure 2b shows the plots. $c_{\epsilon ,1}$ and $c_{\epsilon ,2}$ were determined by calculating the best fit lines of the plots. Figure 2b also shows plots of the best fit lines. The determined values of $c_{\epsilon ,1}$ and $c_{\epsilon ,2}$ are shown in the first row of Table 1. The measurement errors of $c_{\epsilon ,1}$ and $c_{\epsilon ,2}$ were 0.00282 μϵ/MHz and 0.00313 μϵ MHz, respectively.

The increments were determined via calibration with a standard single-mode optical fiber whose strain coefficient was known (Corning^®^ SMF-28^®^). During the experiment, the standard single-mode optical fiber was also wound between the two posts. When the position of the motor-controlled stage was varied, the frequency shift from the standard single-mode optical fiber was measured using the commercial BOTDA. The increment was determined by using the known strain coefficient and the measured frequency shift to calculate Δϵ.

### 3.5. Comparison with References [11] and [12]

Using the experimentally determined values of $c_{T,1}$, $c_{T,2}$, $c_{\epsilon ,1}$ and, $c_{\epsilon ,2}$, δT/δv and δϵ/δv were calculated according to Equations (2) and (3), respectively. δT/δv and δϵ/δv are shown in the first column of Table 1: δT/δv = 4.57 °C/MHz and δϵ/δv = 69.11 μϵ/MHz. These values were compared to those of References [11] and [12]. $c_{T,1}$, $c_{T,2}$, $c_{\epsilon ,1}$ and, $c_{\epsilon ,2}$ reported in References [11] and [12] are shown in the second and third columns, respectively, of Table 1. Using these, δT/δv and δϵ/δv were calculated according to Equations (2) and (3), respectively. δT/δv and δϵ/δv for References [11] and [12] are shown in the second and third columns, respectively, of Table 1. For Reference [11], δT/δv = 12.05 °C/MHz and δϵ/δv = 253.77 μϵ/MHz For Reference [12], δT/δv = 13.25 °C/MHz and δT/δϵ = 299.18 μϵ/MHz. Here, δT/δv and δT/δϵ are 2.63 times and 3.67 times lower, respectively, than for Reference [11]. Here, δT/δv and δT/δϵ are 2.90 times and 4.33 times lower, respectively, than for Reference [12].

### 3.6. Proof of Principle

**Figure 3:**
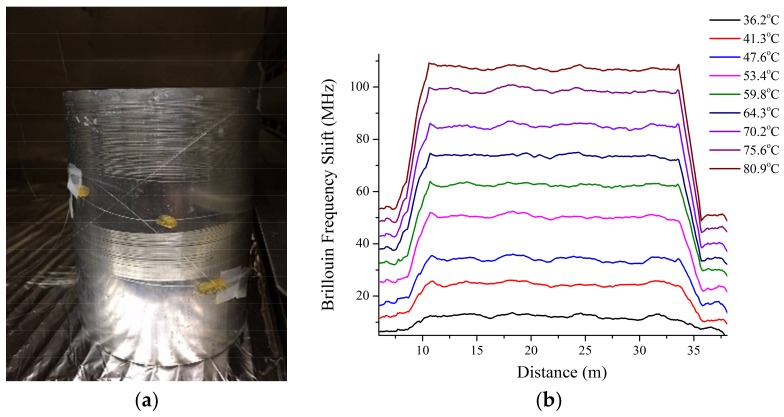
(**a**) Photograph of the Al cylinder with the dual-core samples wound around the cylinder; (**b**) Brillouin frequency shift of one sample at different temperatures and strains caused by the metallic cylinder expansion.

As proof of principle, and to verify the improved accuracy for both temperature and strain measurements, the ΔT and Δϵ of a thermal expansion of a hollow metal cylinder were sensed using the two-core fiber. The metal cylinder was made of aluminum alloy 6063-T5. The diameter of the cylinder is 12 cm with a wall thickness of 3 mm. Two-core fibers with length of 20 meters were tightly wound around the metal cylinder, which is shown in Figure 3a. Cores 1 and 2 of the two-core fiber were probed by the commercial BOTDA via APC patch cables that were fusion spliced to the samples. Although the two-core fiber presented challenges to interrogate using commercially off-the-shelf fiber-connecting devices, this technical difficulty can be readily resolved by using 3D fan-in and fan-out waveguide devices. Using ultrafast laser direct waveguide writing, appropriately low-loss waveguide fan-in and fan-out devices can be readily fabricated in silica glass to connect two standard single-core fibers to the dual-core fibers, and therefore facilitate fiber sensor interrogation [16]. The metal cylinder was placed in an oven. At elevated temperatures, the expansion of the metal cylinder will exert strain on the fiber given the larger thermal expansion coefficient of aluminum alloy compared to that of silica fiber. Given the well-known material properties of the aluminum alloy and its simple geometry, the strain induced on the fiber can be precisely determined. The temperature of the oven was changed from the ambient temperature to 90 °C in increments of approximately 5 °C. For each increment, $\Delta \nu_{1}$ and ${\Delta \nu }_{2}$ were measured using the commercial BOTDA. A thermocouple was placed in the oven to verify the temperature changes of the metal cylinder. The strain changes of the metal cylinder due to thermal expansion were calculated using a finite element analysis (FEA) method (COMSOL). The Brillouin frequency shifts vs temperature along the 20-m section of the fiber of Core 1 are presented in Figure 3b. The measurement was acquired with 1-m spatial resolution and was 5 point-averaged to yield a smooth profile. 

**Figure 4:**
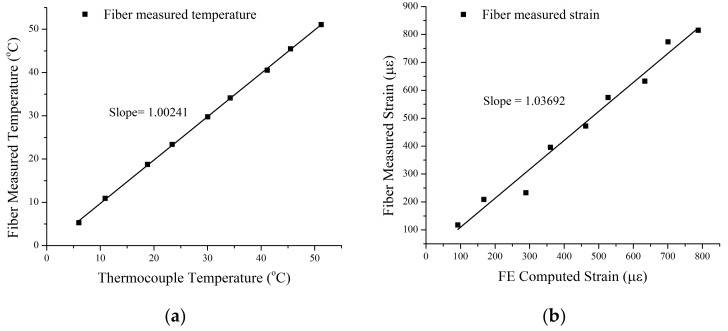
Measurements obtained from dual-core samples for (**a**) temperatures plotted against thermocouple-measured temperatures, and (**b**) strains versus finite-element computed strains. The deviation of these slopes from unity indicates the system error.

For each measurement, temperature and strain measured by the two-core fibers were calculated using the matrix formulation (Equation (1)) from the measured Brillouin frequency shifts $\Delta \nu_{1}$ and $\Delta \nu_{2}$ from Core 1 and Core 2. These results are graphically presented in Figure 4, while fiber-measured temperature and strain are plotted against the temperature measured by the thermal coupler and FEA simulation.

Slopes of linear fits of the temperature and strain calibration figure presented in Figure 4 yields 0.24% system error for temperature measurements and 3.7% system error for strain measurements. The error with the strain measurement is relatively higher than that of temperature. This is due to the fact that the dual core fibers are 13–18 times more sensitive to temperature changes (MHz/°C) than strain changes (MHz/μϵ), as previously depicted in Figure 2. To better illustrate the measurement accuracy at different temperature and strain ranges, experimental data presented in Figure 4 is also presented in Table 2, while dual-core fiber-measured temperature and strains are compared with calibration measurements and calculations from temperatures ranges between 36.1 °C to 81.3 °C. On averages, the two-core fiber yielded 1.7% measurement error for temperatures and 10.7% average error for strain measurements. The large measurement errors are mostly contributed to by those measurements performed at lower temperatures of below 50 °C with lower thermal expansion-induced strains. At higher temperatures of above 50 °C, the average measurement errors reduce to 0.45% and 5.3%, respectively.

## 4. Conclusions

In conclusion, a multicore optical fiber that had two cores was fabricated. The differences between the cores’ temperature and strain coefficients were such that temperature (strain) changes could be discriminated with error amplification factors of 4.57 °C/MHz (69.11 μϵ/MHz), which is 2.63 (3.67) times lower than previously demonstrated. As proof of principle, using the multicore optical fiber and a commercial BOTDA, the temperature (strain) changes of a thermally expanding metal cylinder were discriminated with an average error of 0.24% (3.7%).

Here, it was assumed that the measurement errors of $c_{T,1}$, $c_{T,2}$, $c_{\epsilon ,1}$ and, $c_{\epsilon ,2}$ were negligible. A theoretical analysis of error amplification factors that is more complete than Equations (1)–(3) should account for non-negligible measurement errors of $c_{T,1}$, $c_{T,2}$, $c_{\epsilon ,1}$ and, $c_{\epsilon ,2}$ [13]. However, the measurement errors of $c_{T,1}$, $c_{T,2}$, $c_{\epsilon ,1}$ and, $c_{\epsilon ,2}$ reported here and in References [11] and [12] were a few percent. When using a multicore optical fiber for BOTDA, strain due to bending must be taken into account [11]. However, here, it was assumed that strain due to bending was uniform over the length of the multicore optical fiber and negligible. 

Here, temperature and strain resolutions are not reported. Reporting of strain and temperature resolutions may be considered arbitrary, because temperature and strain resolutions depend on many experimental factors, including launched power, optical fiber length, and measurement time. Because different works utilize differing experimental factors, temperature and strain resolutions may not be comparable. In contrast, error amplification factors are comparable because, for a given set of experimental factors, error amplification factors quantify the difference between the abilities of two multicore optical fibers to discriminate temperature from strain. 

It may be of interest to investigate the use of other types of multimode and multicore optical fibers to discriminate temperature and strain in BOTDA, e.g., those with elliptical cores [17,18,19,20].

## Figures and Tables

**Table 1:** Temperature and strain coefficients ($c_{T,1}$, $c_{T,2}$, $c_{\epsilon ,1}$, and $c_{\epsilon ,2}$) and error amplification factors (δT/δv and δϵ/δv) (first column) in this study; (second column) Reference [11]; and (third column) Reference [12], as described in the text.

	Here	Reference [11]	Reference [12]
$c_{T,1}$ [MHz/°C]	0.9710	1.0300	1.1500
$c_{T,2}$ [MHz/°C]	0.9593	1.0800	1.0500
$c_{\epsilon ,1}$ [MHz/μϵ]	0.0532	0.0517	0.0486
$c_{\epsilon ,2}$ [MHz/μϵ]	0.0729	0.0485	0.0489
δT/δv [°C/MHz]	4.57	12.05	13.25
δϵ/δv [μϵ/MHz]	69.11	253.77	299.18

**Table 2:** (First column) Temperature of metal cylinder as measured by thermocouple; (second column) temperature of metal cylinder as measured using the multicore optical fibers; (third column) strain exerted by metal cylinder as determined by FE analysis; and (fourth column) strain exerted by metal cylinder as measured using the multicore optical fibers as described in the text.

Temperature [°C] (Thermocouple)	Temperature [°C] (Multicore Optical Fiber)	Strain [µϵ] (FE Analysis)	Strain [µϵ] (Multicore Optical Fiber)
36.10	35.39	92.37	117.51
41.00	41.01	167.80	208.72
48.90	48.85	289.41	233.07
53.50	53.49	360.22	395.28
60.15	59.87	462.59	471.80
64.35	64.21	527.25	574.16
71.25	70.63	633.47	632.45
75.60	75.57	700.43	773.47
81.30	81.16	788.18	815.03
